# Cross-linked hyaluronic acid gel inhibits metastasis and growth of gastric and hepatic cancer cells: *in vitro* and *in vivo* studies

**DOI:** 10.18632/oncotarget.11739

**Published:** 2016-08-31

**Authors:** Ting Lan, Ji Pang, Yan Wu, Miaolin Zhu, Xiaoyuan Yao, Min Wu, Hai Qian, Zhenyu Zhang, Jizong Gao, Yongchang Chen

**Affiliations:** ^1^ Department of Physiology, School of Medicine, Jiangsu University, Zhenjiang City, Jiangsu Province, China; ^2^ Department of Obstetrics and Gynecology, Beijing Chao-Yang Hospital of Capital Medical University, Beijing, China; ^3^ R&D Department, BioRegen Biomedical (Changzhou) Co., Ltd., Changzhou, China; ^4^ Basic Medical Department, Changchun Medical College, Changchun City, Jilin Province, China

**Keywords:** cross-linked hyaluronic acid gel (CHAG), inhibition, migration, growth, cancer cell

## Abstract

Cross-linked hyaluronic acid gel (CHAG) has been used to prevent postoperative adhesion of abdominal tumorectomy. However, its effect on tumor cells is still unknown. This paper was designed to investigate the effect of CHAG on metastasis and growth of tumor cells. Migration and invasion assays, Western blotting, pull down assay, siRNA interference, and nude mice implantation tumor model were applied in this study. The results of *in vitro* experiments with gastric cancer cell line AGS and hepatic cancer cell line HepG2 showed that CHAG inhibited the migration and invasion activities, the MAPK and PI3K/Akt mediated signaling, the activation of small G proteins Rac1 and RhoA, and the expression of MMPs and PCNA initiated by EGF, through blocking the activation of EGFR. CHAG also had inhibitory effect on activation of other membrane receptors, including integrin and VEGFR. When the expression of hyaluronic acid receptors (CD44 or RHAMM) was interfered, the above inhibitory effects of CHAG still existed. *In vivo* experimental results showed that CHAG suppressed colonization, growth and metastasis of gastric cancer cell line SGC-7901 in peritoneal cavity of nude mice. In conclusion, CHAG had inhibitory effect on tumor cells, through covering cell surface and blocking the interaction between extracellular stimulative factors and their receptors.

## INTRODUCTION

Tumorectomy is one of the effective therapies for tumors with suitable stages. However, there are primarily two challenges which needed to be solved for performing tumorectomy. Post-operational adhesion is the first one. The patients with adhesion may have chronic pain and bowel abstraction, and the life quantity of them will be made worse. The second one is tumor metastasis, which is one of the main causative factors for poor prognosis and short survival time of the patients undergoing tumorectomy [1]. Postoperative adhesion in peritoneal cavity happens in more than 93% patients received abdominal tumorectomy [2]. The peritoneal cavity is also a well-known metastatic site for intra-abdominal malignancies of several organs, such as stomach, liver, colon, pancreas and rectum [3]. Therefore, prophylactic application of anti-adhesion agentia has been proposed. Moreover, if the adhesion preventive material has anti-tumor effect, it will be more suitable to the application in these patients.

Hyaluronic acid (HA), either native or crosslinking modified, has been broadly used to prevent postoperative adhesion with varies level of successes [4–6]. HA is a non-sulfated glycosaminoglycan consisting of repeated disaccharide units (a-1,4-D-glucuronic acid and β-1,3-*N*-acetyl-D-glucosamine) and presents in all connective tissues as a major constituent of extracellular matrix. HA has been reported with unique role in wound healing [7, 8]. Cluster designation 44 (CD44) and receptor for hyaluronic acid mediated motility (RHAMM) are the receptors of HA. Through binding with these receptors, HA could regulate cell biological activities by activating several signaling pathways, including the transforming growth factor β (TGF-β) mediated, Rho GTPase mediated, and focal adhesion kinase (FAK) mediated pathways [9–11]. In some cancers, HA levels were correlated well with malignancy and poor prognosis. Hence, HA is often identified as a tumor marker for some cancers and used to monitor the progression of the diseases [12, 13].

Research data have shown that HA with different molecular weight/size had different functions. Most studies indicated that HA with low molecular weight promoted tumor development while HA with high molecular weight had opposite effect [14–16]. In clinical, native HA has been used for anti-adhesion after surgery with unsatisfactory results. The fluid feature and rapid degradation of native HA (usually within 48 hours *in vivo*) may contribute to the primary reasons for the failure. However, crosslinking modification is an effective way to enhance the viscosity of HA and reduce the degradation of it, causing the formation of HA hydrogel. This gel can cover the traumatized tissue surface during the critical period of wound healing and prevent adhesion [17]. Clinical study showed that cross-linked HA gel (CHAG) could significantly reduce adhesion in abdominopelvic cavity after gynecological laparoscopic surgeries [18]. However, there is still a lack of information about whether CHAG is safe enough for preventing postoperative adhesion of peritoneal tumorectomy. Or in other words, the effect of this gel on tumor metastasis and growth is not clear while it is applied in preventing post-operative adhesions of tumorectomy. The main aim of this study was to evaluate the effect of CHAG on cancer cell growth and metastasis and to explore the related action mechanism via *in vitro* and *in vivo* experiments.

## RESULTS

### CHAG inhibits basic and EGF-induced migration and invasion activities of gastric and hepatic cancer cells

**Figure 1:**
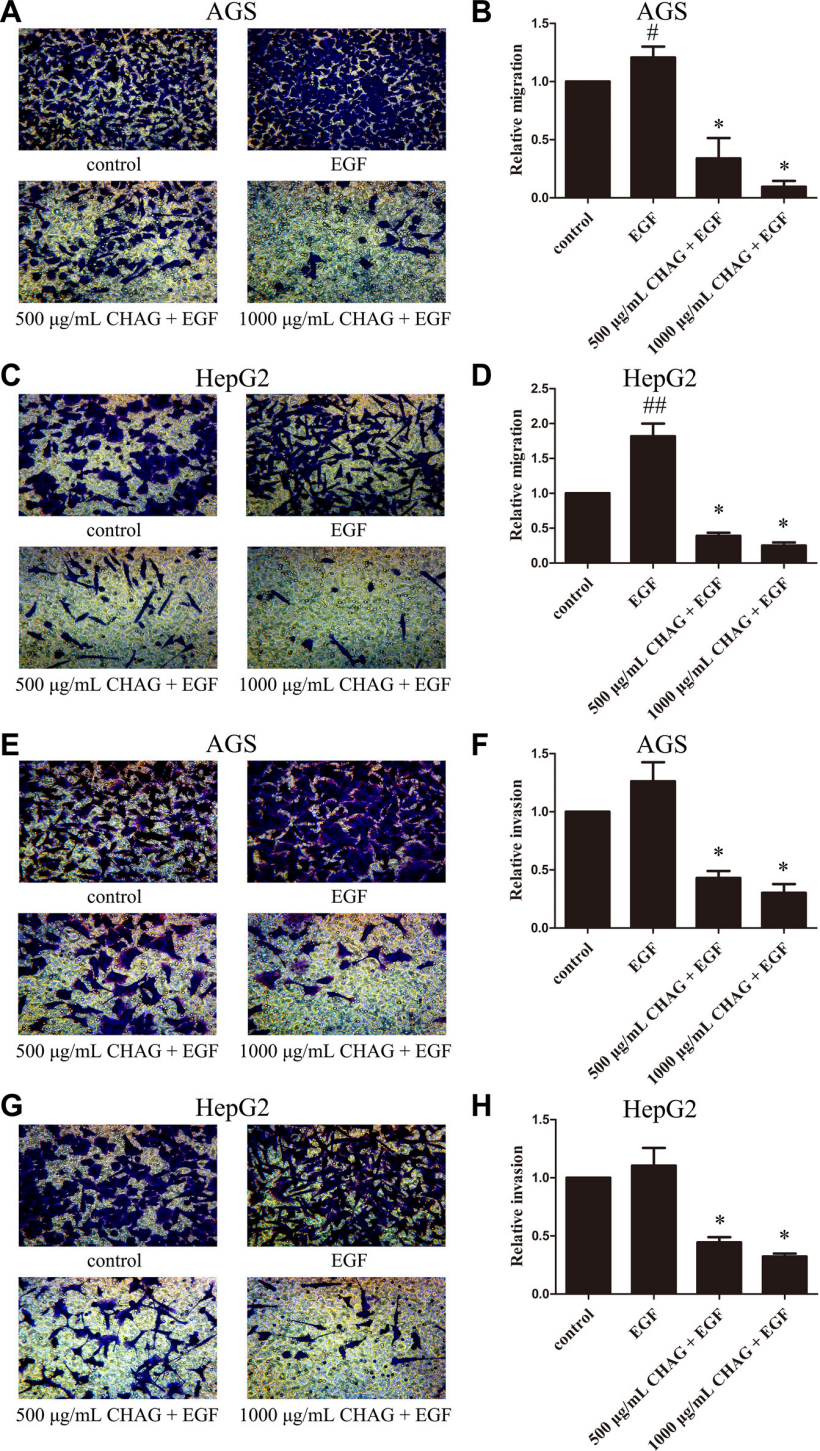
CHAG inhibits migration and invasion activities of gastric and hepatic cancer cells (**A**–**D**) Migration activity of AGS and HepG2 cells. The cells were serum starved overnight, and then divided into Control, EGF, 500 μg/ml CHAG + EGF, and 1000 μg/ml CHAG + EGF groups. In the EGF group, the cells were treated with EGF (100 ng/ml). In the CHAG+ EGF groups, the cells were treated with CHAG (500 μg/ml and 1000 μg/ml respectively) and EGF (100 ng/ml). The migration time was 12 h. (**E**–**H**) Invasion activity of AGS and HepG2 cells. Cell treatments were same to migration assay, except the invasion time was 24 h. A, C, E, and G were representative images of migrated or invaded cells stained by Giemsa (×200). B, D, F, and H were the relative migration or invasion activities of the cells in the corresponding groups. The data shown were the means ± SD from 5 independent experiments, each performed in duplicate. (^#^*P* < 0.05, ^##^*P* < 0.01, compared with control group; *P < 0.01, compared with EGF group).

The results of Trans-well migration and invasion assays showed that CHAG with concentrations of 50 μg/ml, 125 μg/ml, 250 μg/ml, 500 μg/ml and 1000 μg/ml inhibited the basic migration and invasion activities of both AGS and HepG2 cells, with a dosage-dependent pattern (Figure S1). Furthermore, when the migration and invasion activities of AGS and HepG2 cells were stimulated by EGF treatment (100 ng/ml, 12 h), CHAG at the concentrations of 500 μg/ml and 1000 μg/ml significantly inhibited the increase of migration and invasion activities induced by EGF treatment (Figure 1). These results indicated that CHAG had inhibitory effect on both the basic and the EGF-induced migration and invasion activities of AGS and HepG2 cells.

### CHAG inhibits colonization and growth of gastric and hepatic cancer cells in peritoneal cavity of nude mice

**Figure 2:**
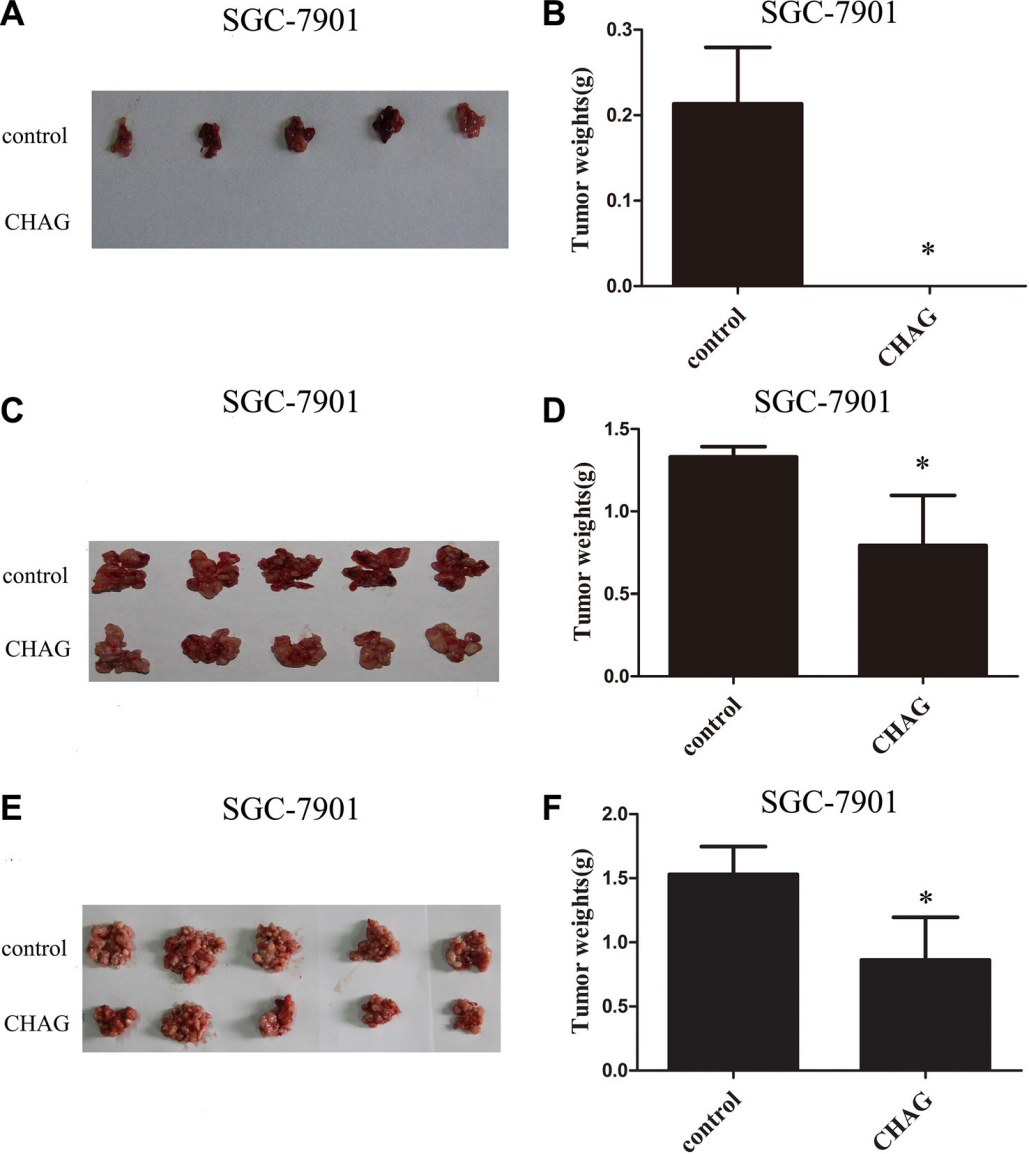
CHAG inhibits colonization and growth of gastric cancer cells in peritoneal cavity (**A** and **B**) The inhibition of CHAG on the colonization of gastric cancer cells in peritoneal cavity. Ten million SGC-7901 cells (suspended in 400 μl PBS) with or without CHAG (500 μg/ml) were injected into peritoneal cavity of nude mouse. Twenty five days later, the mice were executed, the tumors were excised, and the weights of the tumors of the different groups were calculated. (**C** and **D**) The inhibition of CHAG on the early growth of gastric cancer cells. Ten million SGC-7901 cells suspended in 400 μl PBS were injected into peritoneal cavity of nude mice. Two hours late, 400 μl CHAG solution (at concentration of 500 μg/ml) was injected into the cavity once only. The mice were fed normally for 8 weeks and then were executed and the tumors were collected and weighed. (**E** and **F**) The inhibition of CHAG on mid-term growth of gastric cancer cells. The cells were given to the mouse same as described in (C and D) Seven days later, 400 μl CHAG solution (at concentration of 500 μg/ml) was injected into peritoneal cavity of the mouse and the injection was repeated once a week. After 7 weeks, the mice were executed, the tumors were collected and weighed. A, C, and E were images of tumors from the mice in control and CHAG groups. B, D, and F were results of weight analysis of the tumors in corresponding group. The data shown were means ± SD. (*P < 0.01, compared with the control group).

In nude mouse transplantation tumor model, co-injection of CHAG (500 μg/ml) together with transplanted cancer cells completely inhibited the formation of transplantation tumor of SGC-7901 gastric cancer cells (Figure 2A and 2B) and dramatically decreased the weight of transplantation tumor of HepG2 hepatic cancer cells (Figure S2A and S2B). These results indicated that CHAG had inhibitory effect on the attachment/colonization of the cancer cells in peritoneal cavity.

To investigate the effect of CHAG on the early growth of cancer cells, the nude mice were given a one-time peritoneal cavity injection of CHAG (200 μg per mouse, diluted in 400 μl PBS, with a concentration of 500 μg/ml) 2 hours after intra-peritoneal implantation of SGC-7901 gastric cancer cells. To investigate the effect of CHAG on the mid-term growth of transplanted cancer cells, the nude mice were given the first intra-peritoneal cavity injection of CHAG (200 μg per mouse, 500 μg/ml) at the 7th day after the cancer cell implantation and then the injection was repeated weekly for 7 weeks. Both injections significantly decreased the weight of transplantation tumors of SGC-7901 cells (Figure 2C, 2D, 2E and 2F). With HepG2 cells, the experiment of inhibition on early growth was performed and the result was similar to those of SGC-7901 cells (Figure S2C and S2D). These results demonstrated that CHAG inhibited both early growth and mid-term growth of transplanted cancer cells.

### CHAG inhibits the activation of cell membrane receptors of gastric and hepatic cancer cells

**Figure 3:**
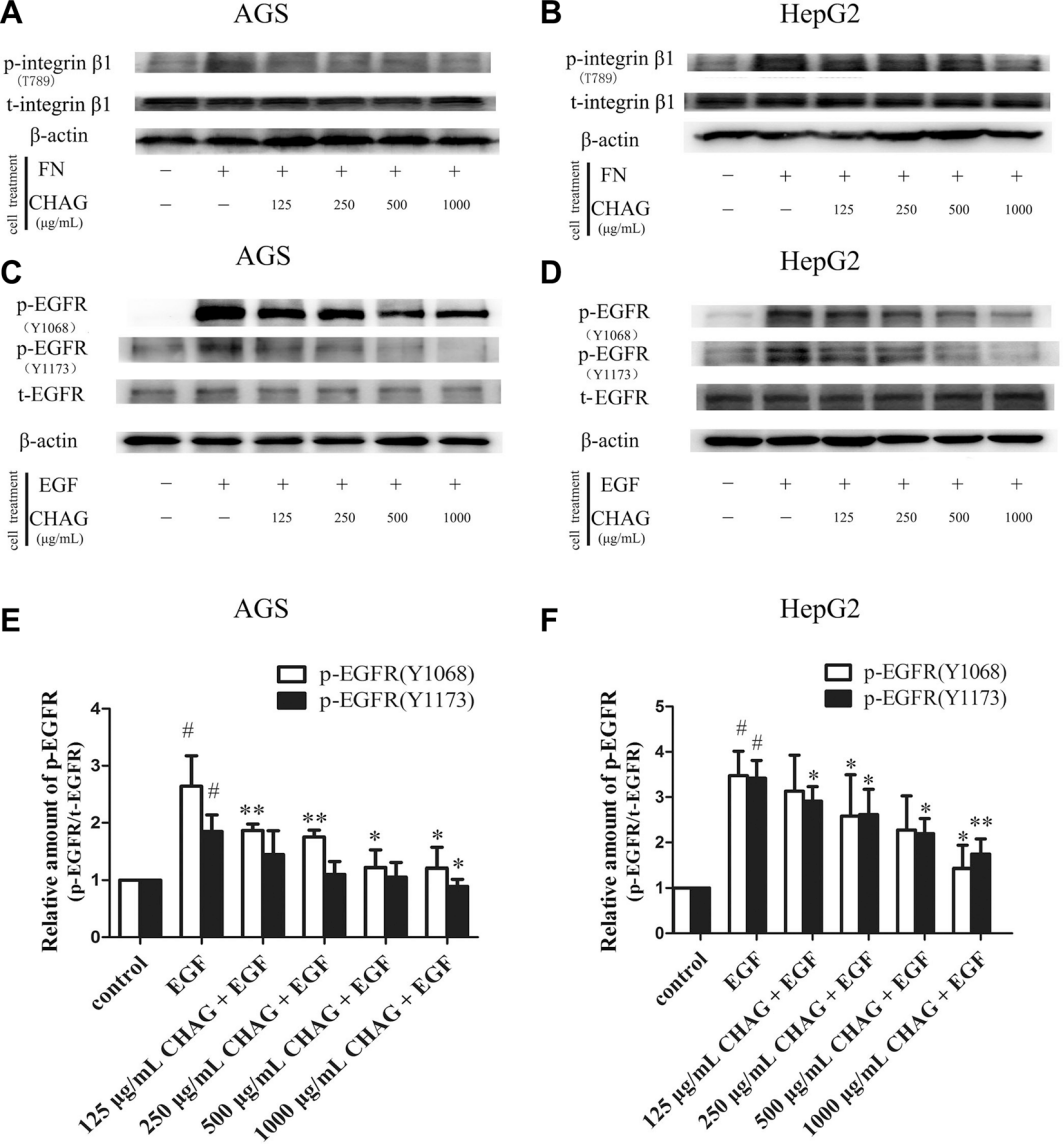
CHAG inhibits activation of membrane receptors in gastric and hepatic cancer cells (**A** and **B**) The inhibition of CHAG on phosphorylation/activation of Integrin β1 in AGS cells and HepG2 cells. The cells were serum starved overnight and treated with fibronectin (FN, 1 μg/ml) for 15 min, or with various CHAG solutions (at concentrations of 125, 250, 500, 1000 μg/ml respectively) for 1 h and then with FN (1 μg/ml) for 15 min. (**C** and **D**) The inhibition of CHAG on phosphorylation/activation of EGFR in AGS cells and HepG2 cells. The cells were serum starved overnight and treated with EGF (100 ng/ml) for 5 min, or with various CHAG solutions (at concentrations of 125, 250, 500, 1000 μg/ml respectively) for 1 h and then with EGF (100 ng/ml) for 5 min. A–D were representative Western blotting results of three independent experiments. (**E** and **F**) were results of densitometry analysis of Western blotting results. (^#^*P* < 0.05, compared with control group; *P < 0.05, **P < 0.01, compared with the EGF group).

Integrin is the transmembrane receptor associated with cell movement through bridging cell-cell and cell-extracellular matrix (ECM) interactions. One integrin molecule consists of one α subunit and one β subunit and integrin α5β1 is fibronectin receptor [19]. To investigate the effect of CHAG on the activity of integrin, the cells were treated with fibronectin and CHAG, and the change of phosphorylation of integrin β1 was detected by Western blotting. The results showed that treatment with fibronectin (1 μg/ml, 15 min) caused obvious increase of phosphorylation of integrin β1. Pre-treatment with CHAG (1000 μg/ml, 1 h) effectively inhibited fibronectin-induced phosphorylation of integrin β1 (Figure 3A and 3B). These results indicated that CHAG could inhibit fibronectin-induced activation of integrin α5β1.

Other cell surface receptors studied in this experiment included EGFR and VEGFR, which were receptor tyrosine kinases (RTKs) associated with tumor progression. Western blotting with antibodies against Tyrosine 1068 (Tyr1068) or Tyrosine 1173 (Tyr1173) phosphorylated EGFR was applied to detect the phosphorylation/activation of EGFR. The result demonstrated that EGF treatment (100 ng/ml, 5 min) led to significant increase of Tyr1068 and Tyr1173 phosphorylation of EGFR, and pre-treatment with CHAG (1000 μg/ml, 1 h) efficiently hindered the EGF-induced phosphorylation of EGFR (Figure 3C–3F), indicating that CHAG inhibited EGF-induced activation of EGFR. Furthermore, CHAG could also inhibit VEGF-induced phosphorylation/activation of VEGFR-2 (Figure S3).

### CHAG inhibits cellular activities downstream of membrane receptors

**Figure 4:**
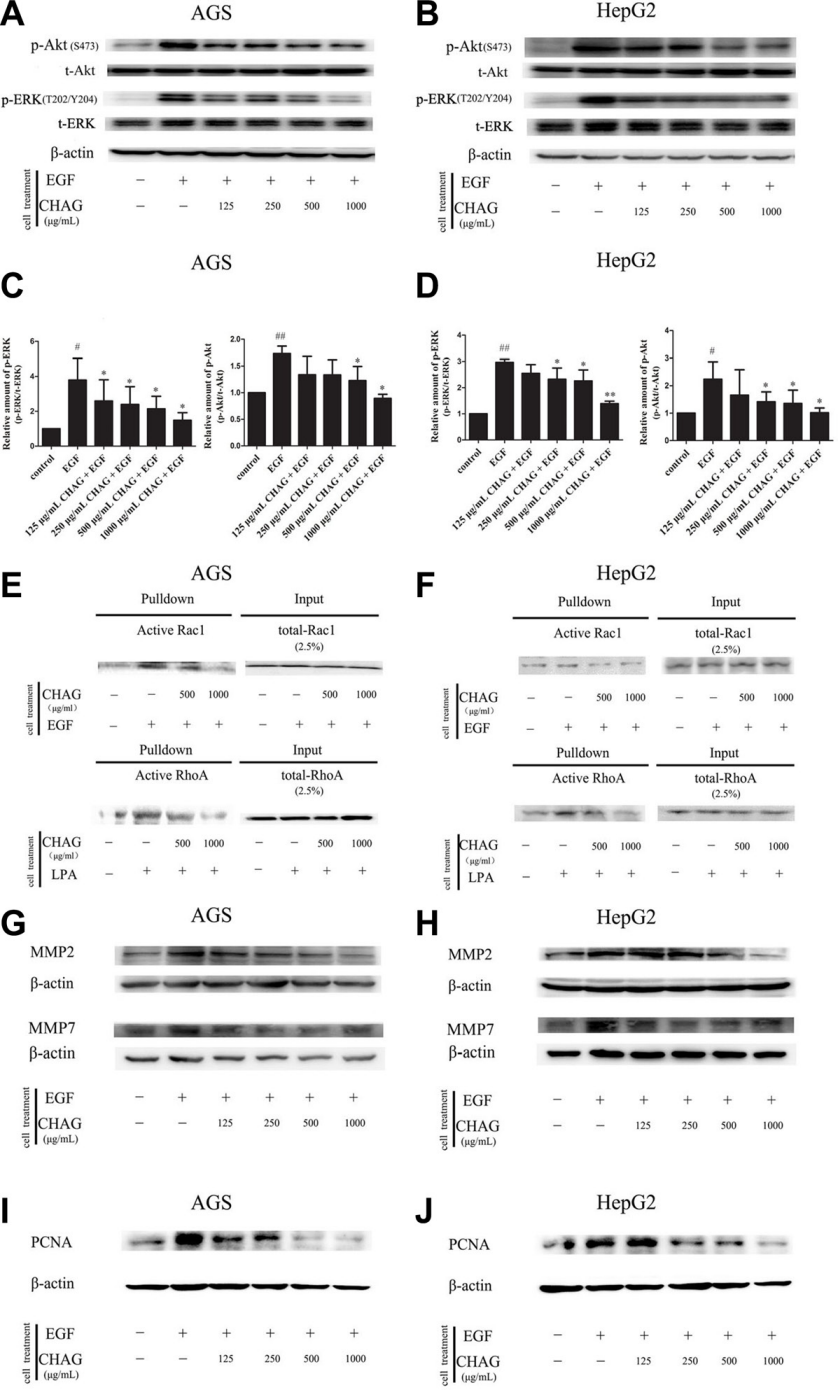
CHAG blocks the activation of downstream signaling molecules of EGFR and inhibits EGF- induced expression of MMPs and PCNA (**A**–**D**) The inhibition of CHAG on the phosphorylation/activation of Akt and ERK in AGS cells and HepG2 cells. The cells were treated same as in Figure 3 (Panel C). The cellular lysates were subjected to Western blotting with antibodies against phosphorylated Akt (p-Akt) or phosphorylated ERK (p-ERK). Total Akt (t-Akt), total ERK (t-ERK) and β-actin were detected as loading control. A and B were the representative Western blotting results of three independent experiments. C and D were results of densitometry analysis of the corresponding Western blotting results. (^#^*P* < 0.05, ^##^*P* < 0.01, compared with control group; *P < 0.05, **P < 0.01 compared with EGF group). (**E** and **F**) CHAG blocked the activation of Rac1 and RhoA in AGS and HepG2 cells. For detection of Rac1 activation, the cells were serum starved overnight, treated with EGF (100 ng/ml, 5 min), or with CHAG solutions (at concentrations of 500, 1000 μg/ml respectively) for 1 h and then with EGF (100 ng/ml, 5 min); For detection of RhoA activation, the cells were serum starved overnight, treated with LPA (1 μM, 5 min), or with CHAG solutions (at concentrations of 500, 1000 μg/ml respectively) for 1 h and then with LPA (1 μM, 5 min). The level of active Rac1 or RhoA was analyzed by ‘‘Pull-down” method. The results were representatives of three independent experiments. (**G** and **H**) Detection of the expression of MMP2 and MMP7 in AGS and HepG2 cells by Western blotting. In EGF group, the cells were treated with EGF (100 ng/ml, 24 h). In the CHAG + EGF groups, the cells were treated with CHAG at various concentrations (125, 250, 500, 1000 μg/ml respectively) and EGF (100 ng/ml) for 24 h. The cells were harvested and the lysates were subjected to Western blotting with anti-MMP2 and anti-MMP7 antibodies. (**I** and **J**) Western blotting detection of the expression of PCNA in AGS and HepG2 cells. The cells were treated same as described in panel G and H, and the lysates were probed by Western blotting with anti-PCNA antibody. The results were representatives of three independent experiments.

Western blotting results showed that EGF treatment (100 ng/ml, 5 min) caused significant increase of phosphorylation/activation of Akt and ERK, which were main signaling components downstream of EGFR. Treatment with CHAG (1000 μg/ml, 1 h) inhibited the stimulating effect of EGF on the activation of these signaling components, confirming the inhibition of CHAG on EGF/EGFR initiated signal transductions (Figure 4A, 4B, 4C, and 4D).

“Pull-down” assay was performed to detect the inhibitory effect of CHAG on activation of small G protein Rac1 and RhoA. The results showed that treatment with EGF (100 ng/ml, 5 min) or LPA (1 μM, 5 min) increased the amount of GTP-bound/active Rac1 or RhoA respectively. Pre-treatment with CHAG (1000 μg/ml, 1 h) efficiently restrained the stimulating effects of EGF and LPA on the activation of the small G proteins (Figure 4E and 4F).

In addition, Western blotting results showed that the expressions of matrix metalloproteinase 2 (MMP2) and metalloproteinase 7 (MMP7) and proliferating cell nuclear antigen (PCNA) were increased by EGF treatment (100 ng/ml, 24 h). Applying CHAG (500 or 1000 μg/ml) with EGF at the same time efficiently inhibited the stimulating effect of EGF on the expressions of MMPs and PCNA (Figure 4G–4J). These results indicated that CHAG could inhibit the expression of migration and proliferation related proteins.

### The tumor-inhibitory effect of CHAG is not related to its binding with HA receptors

**Figure 5:**
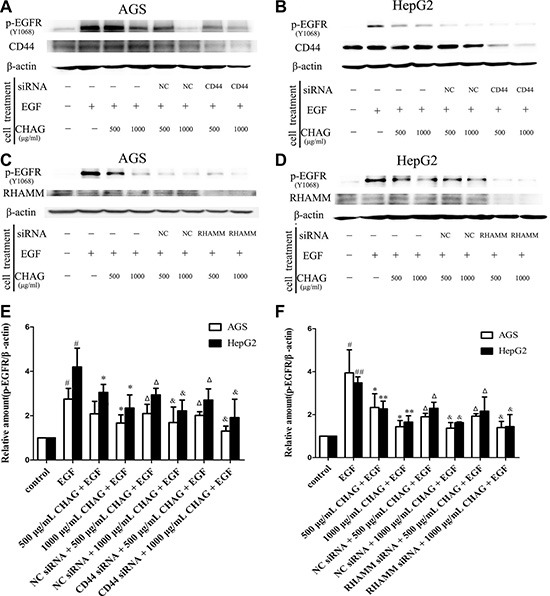
Interference of expression of HA receptors does not affect the inhibition of CHAG on cancer cells (**A** and **B**) The influence of expression interference of CD44 on the inhibitory effect of CHAG. AGS and HepG2 cells were transfected with CD44 siRNA or control siRNA (negative control, NC) for 36 h. And then in the EGF group, the cells were treated with EGF (100 ng/ml) for 5 min. In the CHAG + EGF and CD44 siRNA + CHAG + EGF groups, the cells were treated with two concentrations of CHAG (500, 1000 μg/ml) for 1 h and then with EGF (100 ng/ml) for 5 min. The cells were harvested and the lysates were subjected to Western blotting with corresponding antibodies. (**C** and **D**) The influence of expression interference of RHAMM on the inhibitory effect of CHAG. The cells were transfected with RHAMM siRNA or control siRNA (negative control, NC) for 36 h, and following treatments were same to panel A and B. The results were representatives of three independent experiments. (**E** and **F**): The results of densitometry analysis of Western blotting results of the corresponding groups. (^#^*P* < 0.05, compared with control group; *P < 0.05, **P < 0.01, compared with the EGF group; Δ*P* < 0.01, compared with the EGF group and *P* > 0.05, compared with 500 μg/ml CHAG + EGF group; & *P* < 0.01, compared with the EGF group and *P* > 0.05, compared with 1000 μg/ml CHAG + EGF group).

siRNA interference technology was applied to down-regulate the expression of HA receptors, including CD44 and RHAMM. When the expressions of CD44 and RHAMM in AGS and HepG2 cells were decreased by siRNA, the inhibitory effect of CHAG on phosphorylation/activation of EGFR still existed (Figure 5). These results suggested that CHAG did not actualize its inhibitory effect through binding with CD44 or RHAMM.

## DISCUSSION

In this study, we carried out both *in* vitro and *in vivo* experiments to investigate the effect of CHAG on migration, invasion, growth and implantation of gastric and hepatic cancer cells. In *in vitro* experiment, the results showed that CHAG was able to inhibit the migration and the invasion activities of gastric and hepatic cancer cells. In *in vivo* study, we investigated whether CHAG might affect colonization and growth of gastric and hepatic cancer cells, using a well-defined intra-peritoneal tumor implantation model. The results showed that CHAG, when administrated through injection with the cancer cells at the same time, effectively suppressed the colonization of the cancer cells in peritoneal cavity. When the cancer cells were inoculated into peritoneal cavity first and CHAG was injected into the cavity 2 hours or 7 days later, the administrations simulated the application of CHAG in early growth and mid-term growth of the transplanted cells respectively, the growth of the transplantation tumors was also efficiently inhibited. These results confirmed that CHAG had a definite anti-tumor effect when applied both *in vitro* and *in vivo*.

The effect of HA and polymerized HA on tumor cells had been a disputable topic. Some reported data favored the application of them in prevention of adhesion. For example, Sikkink *et al.* found that bio-absorbable HA membrane resulted in a significant reduction of adhesions, but had no obvious impact on the intra-peritoneal tumor implantation and growth in mice and rats [20]. Haverlag *et al.* also reported that HA-based coating solution had no appreciable effect on intra-abdominal tumor growth in rats and mice [21]. The results from Tian *et al.* suggested that high-molecular-mass HA could induce cancer resistance in naked mole rat [22]. However, other reports warned pernicious effect of the materials. For example, Tan *et al.* reported that sodium hyaluronate enhanced tumor metastatic potential *in vitro* and *in vivo*, suggesting that application of sodium hyaluronate to avoid adhesions might potentiate intra-peritoneal tumor growth after colorectal cancer surgery [23]. The above difference of conclusions may be due to that the biological responses triggered by HA depend on the HA polymer length. It was reported that lower molecular weight HA promoted tumor growth [24], while high molecular weight HA (>1,000 kDa) had inhibitory effect on the tumor [22, 25]. Based on the above data, we speculated that as a HA polymer with boundless molecular weight, CHAG might have an anti-tumor effects similar to the high molecular weight HA. In this paper, our speculation has been proved.

As extracellular substance, how does CHAG affect proliferation and metastasis activity of cancer cells? To answer this question, we investigated whether CHAG affected membrane receptor-initiated cell biological activities. The results demonstrated that CHAG treatment efficiently blocked the phosphorylation/activation of EGFR, integrin and VEGFR, inhibited the EGF-induced signaling of MAPK/ERK, PI3K/Akt and Rac1 mediated pathways, and diminished the EGF-induced expression of proliferation and migration related proteins. LPA-induced RhoA activation was also inhibited by CHAG. These results confirmed that CHAG blocked the activation of some cell membrane receptors, inhibited the downstream signal transduction, and finally down-regulated the expression of related proteins, suggesting that blocking the activation of the receptors was the mechanism for CHAG to inhibit the activities of cancer cells.

The next worthy question was “why CHAG had such a wide-range inhibitory effect on the receptors?”. We put forward two assumptions for answering this question. One was that CHAG might specifically bind and cause the activation of HA receptors and then exert its inhibitory effect on other membrane receptors. The other one was that CHAG with sticky property might prevent all of the interactions between the stimulating factors and their receptors by wrapping around the cells. To clarify whether the anticancer effect of CHAG was via binding/activating HA receptors, the cells were transfected with siRNA to decrease the expression of CD44 or RHAMM and the change of inhibitory effect of CHAG was investigated. The results showed that when the expression of CD44 or RHAMM was significantly decreased, the inhibitory effect of CHAG on the activation of EGFR still existed, indicating that the inhibitory effect of CHAG on the EGFR activation was not through binding with and activating HA receptor CD44 and RHAMM. Furthermore, there was no research data indicating the connection between CD44/RHAMM and other membrane receptors such as EGFR, integrin, VEGFR and LPA receptor. Therefore, it was likely that CHAG, owing to its physical sticky property, wrapped the cells and prevented the interaction between stimulating factors and their corresponding receptors, and therefore blocked the activation of the receptors, contributing to the inhibition on migration, invasion and proliferation activities of the cancer cells.

The most noteworthy results of this study came from the *in vivo* experiment with implantation tumor model. The results indicated that when CHAG was administrated together with gastric cancer cells through intra-peritoneal injection, the formation of implantation tumors were completely abolished in gastric cancer cells and dramatically decreased in hepatic cancer cells, indicating the CHAG could efficiently hinder the attachment/colonization of disseminated tumor cells in peritoneal cavity. To investigate the effect of CHAG on the growth of implantation tumors, the gel was administrated 2 hours or 7 days after tumor cell inoculation in peritoneal cavity. Application of CHAG 2 hours after cancer cell inoculation could simulate the period that the detached tumor cells had been implanted and begun to grow in peritoneal cavity while application of CHAG 7 days after cancer cell inoculation could simulate the period of mid-term growth of the implantation tumors. The results showed that CHAG had inhibitory effect on tumor growth in both situations. This confirmed that, except the anti-metastasis effect, CHAG also had anti-proliferation effect on tumor cells. All of these results will have very important clinical significance, and make it safe to use CHAG in clinical tumorectomy for preventing postoperative adhesion.

In summary, our results demonstrated that CHAG could prevent the interaction between stimulating factors and their receptors, block the downstream signal transduction and inhibit tumor progress by wrapping around the cells. This suggests that application of CHAG to prevent post-operative adhesion of tumorectomy may also hinder tumor implantation, growth and metastasis in peritoneal cavity, possessing the effect of killing two birds with one stone.

## MATERIALS AND METHODS

### Cell migration assay

Trans-well plates (Costar, Corning, USA) were used to analyze migration activity of human gastric cancer cell line AGS and human hepatic cancer cell line HepG2 (from Institute of Cell Biology, Shanghai, China), according to the manufacturer's instruction. Briefly, after trypsinization, the cells were suspended in DMEM culture medium (GIBCO, Grand Island, USA) at a concentration of 5 × 10^5^/mL in control groups. In CHAG groups, the cells were suspended in DMEM containing CHAG (From BioRegen Biomedical Co. Ltd, Changzhou, Jiangsu, China) at the same concentration of control group. In epidermal growth factor (EGF, from Sigma, St. Louis, USA) group, EGF (100 ng/ml) was added to the cell suspension to stimulate the migration of the cells. In the upper chamber of the well, 300 μl cell suspension was added. Cell migration to the bottom side of the membrane was induced by 500 μl of DMEM with 10% FBS (GIBCO, Grand Island, USA) in the lower chamber. The migration time was 12 h. At the end of the migration, the cells migrated onto the bottom side of the membrane were stained with Giemsa and then observed and counted under light microscopy.

### Cell invasion assay

Cell invasion assays were performed using the trans-well plates same as described in cell migration assay except that the membrane of the upper chamber was coated with 60 μl of Extracellular Matrix (ECM, 0.125 μg/μl, from Sigma, St. Louis, USA). The cells were treated with CHAG and EGF and seeded into the upper chamber in the same way as for the migration assay. After incubation for 24 h at 37°C, the cells migrated onto the bottom side of the membrane were stained and counted. Subsequent operation was same as for the migration assay.

### *In vivo* study on the tumor inhibition effects of CHAG in a nude mouse tumor transplantation model

This experimental study received full approval from the Institutional Animal Case and Use Committee (IACUC). Specific Pathogen Free (SPF) grade BALB/c nude mice with weights of 8.76 ± 1.34 g were maintained in a SPF barrier system. In the colonization inhibition experiment, 1 × 10^7^ cells of SGC-7901 gastric cancer cell line (from Institute of Cell Biology, Shanghai, China) suspended in 400 μl of PBS or PBS containing CHAG (20 μg per mouse, at a concentration of 500 μg/ml) were implanted into each mouse by intra-peritoneal cavity injection. The mice were bred for 25 days under standard conditions. In the proliferation/growth inhibition experiment, each mouse was given the same amount of cancer cells suspended in PBS. For experiment of early growth inhibition, 400 μl PBS or 400 μl PBS containing CHAG (20 μg per mouse, at concentration of 500 μg/ml) were injected into the peritoneal cavity of the mouse two hours after cancer cell implantation. The animals were normally fed for 8 weeks. For experiment of mid-term growth inhibition, the weekly injection of PBS or PBS containing CHAG were started at the 7th day after cancer cell implantation and repeated for 7 weeks. At the end of the experiment, the animals were euthanized, and the tumors were collected and weighed.

### Western blotting

The differently treated AGS and HepG2 cells were harvested. Protein samples were subjected to SDS-PAGE and membrane transfer was performed following the manufacturer's protocol (Bio-Rad, Hercules, CA). The primary antibodies were incubated over night at 4°C, and the corresponding secondary antibodies (West Grove, PA, USA) were incubated for 1 h at RT, with three washes after each incubation. ECL reagents (Billerica, MA, USA) were used to show the positive bands on the membrane.

### Cell transfection and RNA interference

For transfection, the AGS and HepG2 cells were seeded in six-well plates at a density of 80% confluence and transfected at the following day. Transfection of cells with siRNA for CD44 or RHAMM was performed using Lipofectamin 2000 (Invitrogen, Carlsbad, CA), following the manufacturer's instruction. After 36 h, the cells were treated with CHAG (500, 1000 μg/ml) for 1 h and then with EGF (100 ng/ml) for 5 min. The protein was extracted and detected by Western blotting.

### “Pull-down” analysis of active small G protein RhoA and Rac1

The activity of RhoA was detected with Pull-down method. The cells were treated with CHAG (500, 1000 μg/ml) for 1 h and EGF (100 ng/ml) for 5 min, and then lysed in lysis buffer (25 mM HEPES pH 7.5, 150 mM NaCl, 1% NP40, 10% glycerol, 25 mM NaF, 10 mM MgCl_2_, 0.25% sodium deoxycholate, 1 mM EDTA,1 mM Na_3_VO_4_, 10 mg/ml aprotinin and 10 mg/ml leupeptin). The protein extracts were incubated with Rhotekin-RBD bound to glutathione-agarose beads. The activated RhoA bound to the beads or total RhoA in cell extracts was detected by Western blotting with antibody against RhoA (Santa Cruz Biotechnology, Dallas, USA). The active Rac1 was detected with similar method but with GST-Pak1 protein binding domain (GST-PBD) and antibody against Rac1 (Cell Signaling Technology, Danvers, USA).

### Statistical analysis

All *in vitro* experiments were performed in triplicate for each cancer cell type and each treatment setting. Data are expressed as means ± standard deviation (SD). Statistical significance was performed using a two-tailed ANOVA with SPSS statistical software. Student's *t* test was performed if equal variance was ascertained in two groups by *F* test. A *P*-value of less than 0.05 was considered significant.

## SUPPLEMENTARY MATERIALS FIGURES


