# Supplementary material for: Cross-linked hyaluronic acid gel inhibits metastasis and growth of gastric and hepatic cancer cells: in vitro and in vivo studies
Source: Oncotarget. 2016 Aug 31;7(40):65418–28. doi: 10.18632/oncotarget.11739 (PMC5323165; doi:10.18632/oncotarget.11739)
Supplement: Supplementary file 1 [file oncotarget-07-65418-s001.pdf]

Cross-linked hyaluronic acid gel inhibits metastasis and growth of gastric and hepatic cancer cells: *in vitro* and *in vivo* studies

Supplementary Materials

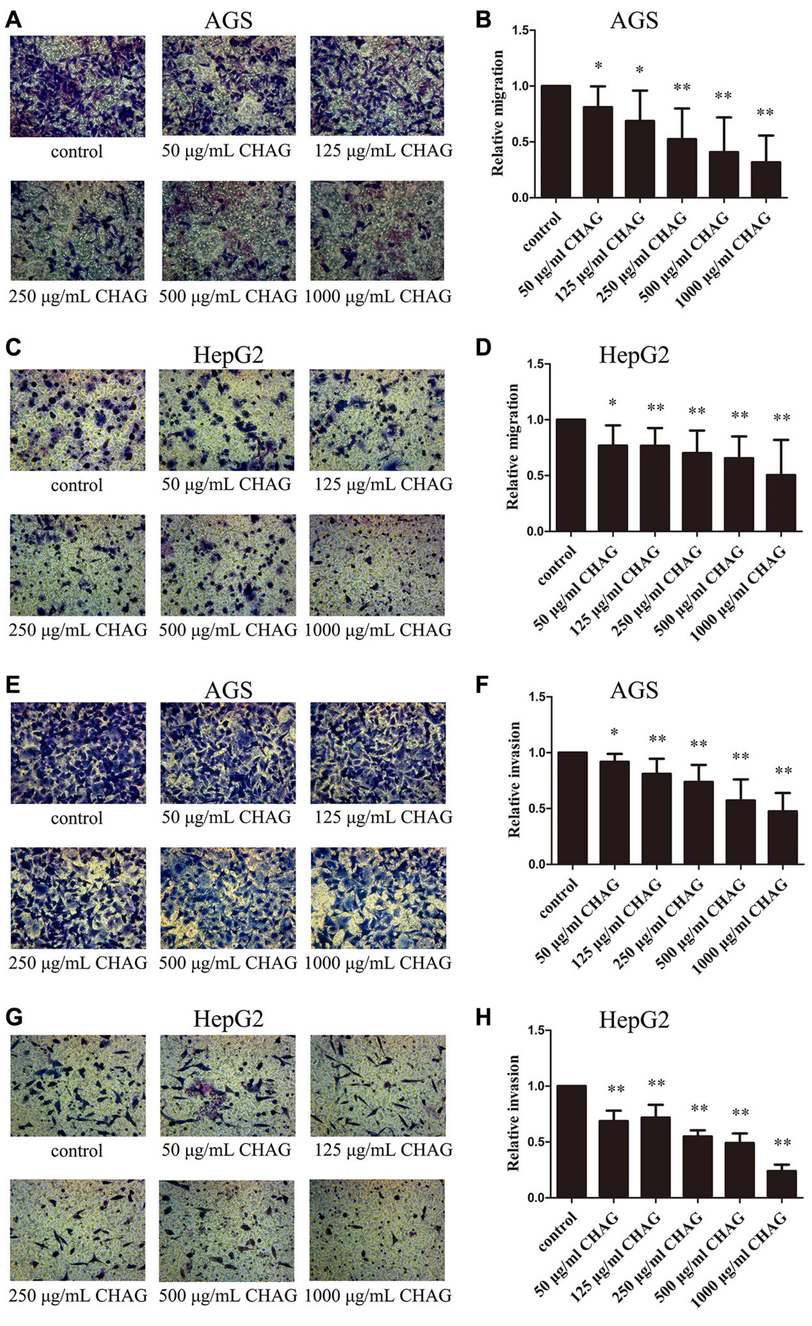

Supplementary Figure S1: CHAG inhibits basic migration and invasion activities of gastric and hepatic cancer cells. (**A–D**) Results of cell migration assay. AGS and HepG2 cells were divided into control group and groups with different concentrations of CHAG (50, 125, 250, 500, 1000 µg/ml), and treated as described in "Cell migration assay" section. The migration time was 12 h. (**E–H**) Results of cell invasion assay. The membrane of the upper chamber of the transwell plate was coated with ECM as described in "Cell invasion assay" section. Both AGS and HepG2 cells were grouped same as in panel A–D and treated as described in "Cell invasion assay" section. The invasion time was 24 h. A, C, E, G were representative figures of migrated cells stained by Giemsa (×200); B, D, F, H were the relative migration activities of number of the corresponding groups. The data shown are the means ± SD from 5 independent experiments, each performed in duplicate. (**$P < 0.01$, *$P < 0.05$, compared with Control group).

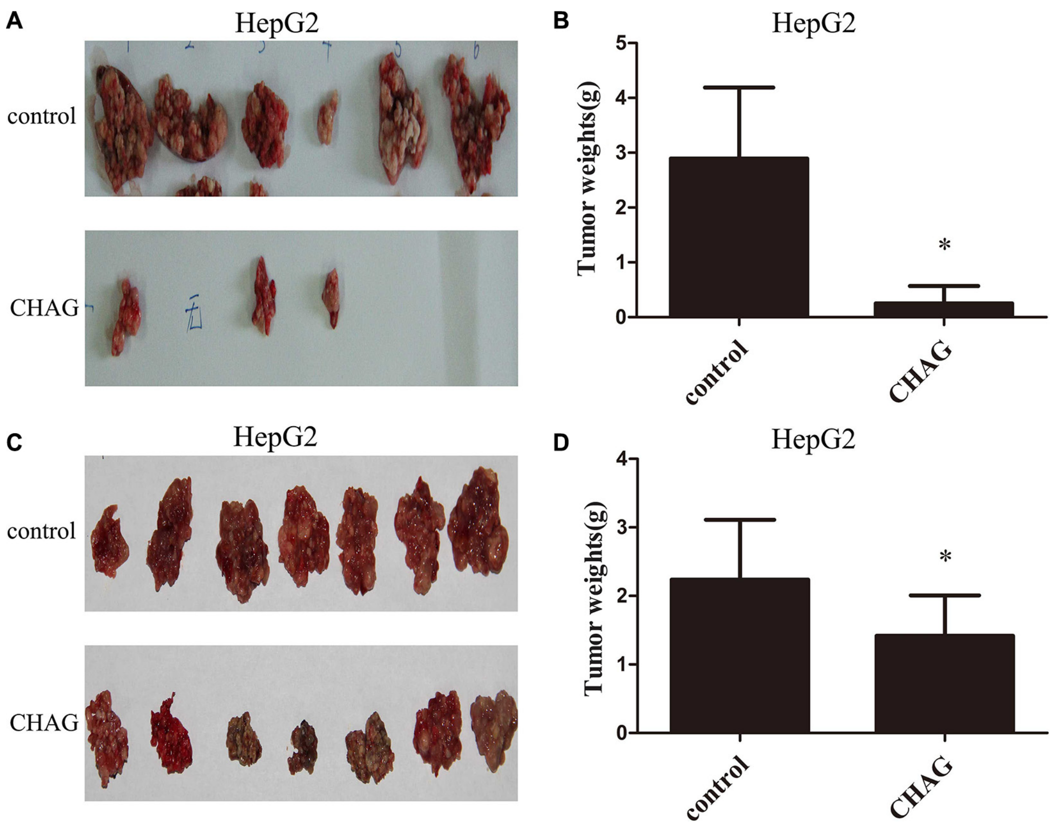

Supplementary Figure S2: CHAG inhibits colonization and growth of hepatic cancer cells in peritoneal cavity of nude mouse. (**A**, **B**) The inhibition of CHAG on the colonization of hepatic cancer cells. Ten million HepG2 cells with or without CHAG solution (at concentration of 500 μg/ml) were injected into peritoneal cavity of nude mouse. Twenty five days later, the mice were executed, the tumors were excised, and the weights of the tumors of the different groups were calculated. (**C**, **D**) The inhibition of CHAG on the early growth of transplanted tumors. Ten million HepG2 cells suspended in 400 μl PBS were injected into the peritoneal at the 2nd hour after inoculation of the cells, CHAG (400 μl, 500 μg/ml) was injected into the peritoneal cavity of the mouse. After being normally fed for 8 weeks, the mice were executed and the tumors were collected and weighed. A, C were the images of tumors from the mice in corresponding groups; B, D were results of weight analysis of the corresponding groups. The data shown were the means ± SD. (**P* < 0.05 compared with the control group).

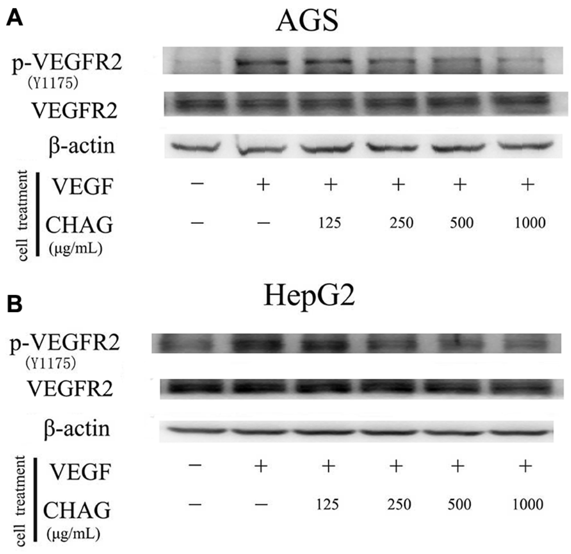

Supplementary Figure S3: CHAG inhibits activation of VEGFR in gastric and hepatic cancer cells. (**A**) The inhibition of CHAG on phosphorylation of VEGFR in AGS cells. The cells were serum starved overnight and treated with VEGF (100 ng/ml) for 5 min, or with various concentrations of CHAG (125, 250, 500, 1000 μg/ml) for 1 h and then with VEGF (100 ng/ml) for 5 min. The cells were harvested and lysed, and the lysates were subjected to Western blotting with antibody against p-VEGFR and VEGFR. (**B**) The inhibition of CHAG on phosphorylation of VEGFR in HepG2 cells. The cells were treated and the lysates were detected same as described in panel A.
